# Hovering hummingbird wing aerodynamics during the annual cycle. I. Complete wing

**DOI:** 10.1098/rsos.170183

**Published:** 2017-08-23

**Authors:** Yonathan Achache, Nir Sapir, Yossef Elimelech

**Affiliations:** 1 TASP—Technion Autonomous Systems Program, Technion-Israel Institute of Technology, Haifa, Israel; 2 Animal Flight Laboratory, Department of Evolutionary and Environmental Biology, University of Haifa, , Israel

**Keywords:** aerodynamics, hovering, hummingbird, leading-edge vortex, animal flight

## Abstract

The diverse hummingbird family (Trochilidae) has unique adaptations for nectarivory, among which is the ability to sustain hover-feeding. As hummingbirds mainly feed while hovering, it is crucial to maintain this ability throughout the annual cycle—especially during flight-feather moult, in which wing area is reduced. To quantify the aerodynamic characteristics and flow mechanisms of a hummingbird wing throughout the annual cycle, time-accurate aerodynamic loads and flow field measurements were correlated over a dynamically scaled wing model of Anna’s hummingbird (*Calypte anna*). We present measurements recorded over a model of a complete wing to evaluate the baseline aerodynamic characteristics and flow mechanisms. We found that the vorticity concentration that had developed from the wing’s leading-edge differs from the attached vorticity structure that was typically found over insects’ wings; firstly, it is more elongated along the wing chord, and secondly, it encounters high levels of fluctuations rather than a steady vortex. Lift characteristics resemble those of insects; however, a 20% increase in the lift-to-torque ratio was obtained for the hummingbird wing model. Time-accurate aerodynamic loads were also used to evaluate the time-evolution of the specific power required from the flight muscles, and the overall wingbeat power requirements nicely matched previous studies.

## 1. Introduction

The Trochilidae is a diverse New World bird family that is highly adapted to nectarivory. A key attribute of hummingbird flight is the ability to sustain hovering for a long period of time, especially during feeding [1,2]. In aerodynamics, hovering is arguably the hardest feat to accomplish as only muscle power is used to support weight [3–5]. The aerodynamic ability to stay airborne while hovering must be preserved throughout the entire annual cycle, including during the energy-demanding period of feather moult, in which wing surface is reduced [6–8]. It is believed that gaps created in the wing during moult may lead to deteriorated aerodynamic capabilities and even failure to hover [9–13]. Therefore, it is important to analyse the aerodynamic loads and the corresponding flow fields throughout the annual cycle, including during flight feather moult, and the ways they may relate to the flyer’s mass and energetics. The present contribution describes our analysis of a complete hummingbird wing. In a second contribution, the same techniques are applied to analyse the aerodynamic loads and flow field over wing models that characterize several flight feather moult stages [14].

Hummingbirds operate at a chord-based Reynolds number (Re) regime of 5000–30 000 [15,16]. While hovering, hummingbirds flap their wings in a nearly horizontal figure eight shape, resembling wing kinematics found in several insect groups [17]. For this reason, it was generally assumed that hummingbirds use similar flow mechanisms as insects, namely, an attached leading-edge vortex [18], despite the large operational Reynolds number difference (insects, Re=10–5000).

In recent years, the ability to analyse flow fields over *in vivo* natural flyers has altered a few common beliefs about the aerodynamics of hummingbirds. Studies of free-flying hummingbirds have found the lift production to be asymmetric between the stroke-halves, where the power stroke, denoted herein as the downstroke, produces up to 75% of the weight support, contrary to insects where wing support is symmetric [19,20]. Moreover, recent flow measurements have also suggested a relatively small vorticity concentration over the leading-edge of a free-flying rufous hummingbird (*Selasphorus rufus*, [19,21]). The authors reported that the intensity of this vorticity is highly variable and corresponded to changes between 0.7 and 26% of the total lift production. Similar vorticity concentrations were also measured over the leading-edge of several rotating, dried hummingbird wings at high angles of attack [22].

While large and attached leading-edge vortices characterize the flow field over insect wings (Re<5000 [23–28]), this coherent vorticity structure was reported to break down beyond an operational Reynolds number of 10 000 [29]. Furthermore, Elimelech & Ellington [30] have shown that in a steady rectilinear flow over a hovering hummingbird wing model, the flow was highly unsteady and a transition process was evident even at Reynolds numbers as low as 5000. Such destruction of the leading-edge vortex may strongly diminish the high lift production which is associated with it [29]. Therefore, to better understand how lift is produced, it is of importance to quantify the geometrical extent and the intensity of the vorticity concentration along the hummingbird wing and its characteristics (steady or unsteady).

To meet the tall order of power requirements during hovering, hummingbirds have developed efficient and effective biomechanical systems [3], among which are their flight muscles, comprising 25% of their body mass [31], a value commonly found in flyers with high vertical take-off speed [32]. In hummingbirds, the mass ratio between the supracoracoideus and pectoralis, the flight muscles corresponding to the upstroke and downstroke, is among the highest found in nature [33]. Typically, the supracoracoideus muscle is about one-fifth of the pectoralis mass, but in hummingbirds the ratio is 1 : 2, most likely to allow the upstroke to provide sufficient weight support [3,33].

Estimations of muscle power requirements and wing performance of hummingbirds have been previously studied, mainly by using quasi-steady models [34,35]. These models allow for an indirect estimation of power requirements of free-flying hummingbirds using wing kinematics and morphologies data [4,31,36]. These methods, however, are prone to large uncertainties. Dried hummingbird wings were also tested, mounted on a constantly rotating propeller rig [16,22], allowing experimental repeatability and the ability to accurately preserve wing geometries and surface textures. However, this approach suffers from poor dynamic range measurements as the aerodynamic loads in the air at biologically relevant Reynolds numbers are in the order of a few grams and are consequently difficult to measure. Dynamically scaled planar wings allow for simplification of the mechanical set-up but pose an inherent bias as they do not represent the exact geometry of the wing [16]. In the light of the disadvantages in previously developed experimental methods, in the study presented herein, we investigate the unsteady mechanisms of a three-dimensionally scanned and dynamically scaled Anna’s hummingbird (*Calypte anna*) wing during the downstroke. By combining high-fidelity, time-accurate aerodynamic loads and flow-field measurements, our aim is to enhance the understanding of the flow mechanisms that develop during the wing’s downstroke and to correlate them to the time-accurate lift, drag and power requirements.

## 2. Material and methods

### 2.1. Model wing design and validation

Within the large family of hummingbirds, Anna’s hummingbird is medium-sized; it has an average adult body length of approximately 100 mm, a wing span of 114–121 mm [30] and an average mass of approximately 4.6 g [37]. Anna’s hummingbird flaps its wings, made mostly of 10 primary flight feathers, at a frequency of about 40 Hz (±5% [20,38,39]).

**Figure 1.**
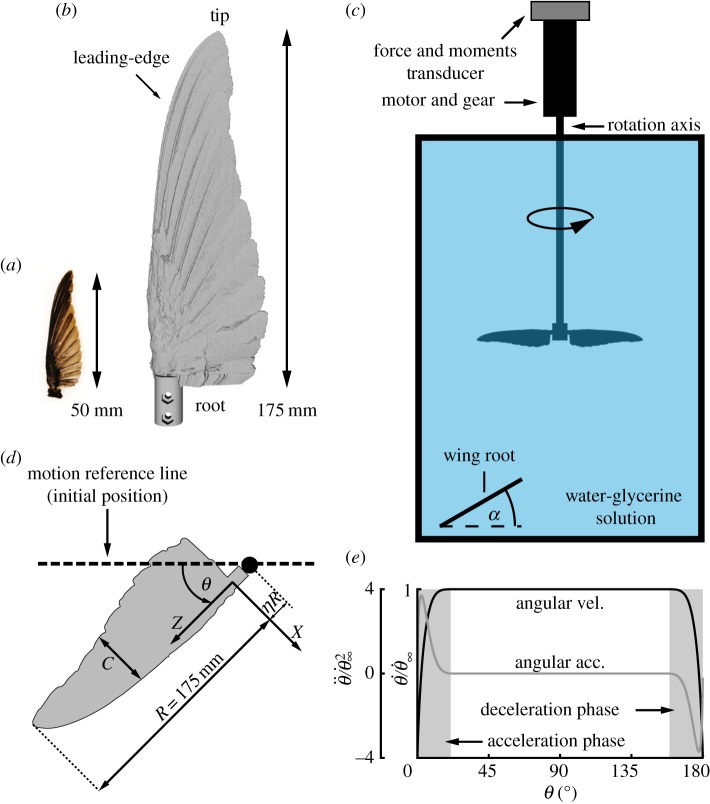
Top view of (*a*) *C. anna*’s dried wing and (*b*) a 3.5 : 1 wing model. (*c*) Schematic illustration of the experimental set-up. The angle of attack, *α*, is defined as the physical angle between the wing’s root chord and the horizontal plane. (*d*) Top view of the wing, depicting the coordinate system. (*e*) The wing motion kinematics, where $\dot{\theta }$ and $\ddot{\theta }$ are the instantaneous angular velocity and acceleration, respectively. ${\dot{\theta }}_{\infty }$ is the angular velocity at mid-downstroke (*θ*=90°).

Based on three-dimensional laser scans of a dried, complete male *C. anna* wing ([30], figure 1*a*), a 3.5:1, dynamically scaled model wing (figure 1*b*) was fabricated using stereolithography (Objet VeroBlack^TM^). Previous studies found that hummingbird wings are rigid, particularly during the downstroke [17,18]. Moreover, scanning electron microscope (SEM) measurements of *C. anna*’s wing showed a physical connection between neighbouring barbs [30], justifying the use of a rigid and non-porous material for the wing model in our experimental scheme presented below. It is also assumed that wing deformation is minimal [40] and does not play a significant role during the downstroke due to special feather arrangement, as opposed to during upstroke and wing reversal. Therefore, only the downstroke is analysed herein. Regarding wing reversal, there is evidence that it contributes additional circulation generated as the wing rotational velocity decays [19]. However, it is believed that higher fidelity results are required in order to adequately quantify this aspect and consequently it will be the subject of a future study.

### 2.2. Experimental methods

To investigate the flow over the Anna’s hummingbird wing, time-accurate measurements of the aerodynamic loads acting on the wing, along with flow field measurements using phase-locked particle image velocimetry (PIV), were conducted. The study was carried out using a pair of wings suspended in a tank (0.6×0.6×1 m) filled with a working fluid (figure 1*c*) that varied between the two sets of experimental set-ups to meet the aerodynamic similarity constraints and their inherent limitations. A controller unit (Technosoft IPOS3604-VX-CAN, Neuchâtel, Switzerland) controlled a servomotor (3257CR32A, Faulhaber, Schönaich, Germany) which rotated the wing pair around a vertical axis (figure 1*c*). Each wing followed stages of initial acceleration, steady rotation and deceleration to model the downstroke of a flapping wing (figure 1*e*). The motion command was subjected to the kinematical constraint of approaching mid-downstroke, where *θ*=90°, at the middle of each wing cycle (*θ* denotes the wing’s angular position, figure 1*d*). These simplifications allowed us to coherently analyse the basic flow mechanisms that developed over the wing at one distinct Reynolds number, avoiding second-order effects. In this study, Re=10 000 and is defined as

2.1 $$\text{Re}=\frac{U_{\mathrm{tip}}^{90^{\circ }}\bar{c}}{\nu },$$

based on the wing tip velocity at mid-downstroke, $U_{\mathrm{tip}}^{90^{\circ }}={\dot{\theta }}_{\infty }R(1+\eta )$, and the mean chord, $\bar{c}$. *R* is one wing span, *η* is the dimensionless distance between the wing’s root and the centre of rotation (figure 1*d*), ${\dot{\theta }}_{\infty }$ is the angular velocity at mid-downstroke (figure 1*e*) and *ν* is the kinematic viscosity of the working fluid (table 1).

**Table 1.** Experimental dimensional data: *ρ* is the working fluid’s specific weight; *ν* is the working fluid’s kinematic viscosity; *T* is the duration of each experimental repetition; *f* is the flapping frequency; ${\dot{\theta }}_{\infty }$ is the angular velocity at mid-downstroke; *R* is one wing span, *R*_2_ and *R*_3_ are the wing radii of second and third moments of area, respectively; *η* is the dimensionless distance between the wing’s root and the centre of rotation (referenced to *R*); $\bar{c}$ is the wing’s mean chord; *S* is the wing area; A is the wing aspect ratio based on *R* and *S*.

	force measurements		flow measurements
*ρ* (kg^−1^ m^3^)	1110		1000
*ν* (cSt)	4.6		1.2
*T* (s)	0.74		2.8
*f* (s^−1^)	0.83		0.21
${\dot{\theta }}_{\infty }\;(\mathrm{rad}\;s^{-1})$	5.23		1.36
*R* (mm)		175	
*R*_2_/*R*		0.56	
*R*_3_/*R*		0.59	
*η*		0.21	
$\bar{c}\;(\mathrm{mm})$		39.37	
*S* (m^2^)		9.12×10^−3^	
A		3.35	

### 2.3. Aerodynamic load measurements

Measuring time-accurate aerodynamic loads at Reynolds numbers of several tens of thousands and below is challenging, because the variation in physical loads can be subtle, requiring a highly sensitive measuring system. Moreover, resolving the time development of the aerodynamic loads requires a reliable mechanical system and high speed data acquisition tools due to the high frequency of the flapping wings. Using dynamic similarity rules, the aerodynamic loads were measured in water–glycerine solutions rather than in air. The 3.5:1 dynamically scaled wing model, together with the high specific weight of the working fluid (water–glycerine) and its viscosity (table 1), allowed for the obtainment of a biologically relevant Reynolds number of 10 000 at a revolving frequency which was in the order of 1 Hz (table 1). In these conditions, the dimensional aerodynamic loads were about five times higher than those that would have been obtained in air, achieving a wider dynamic range for the load measurements.

**Figure 2.**
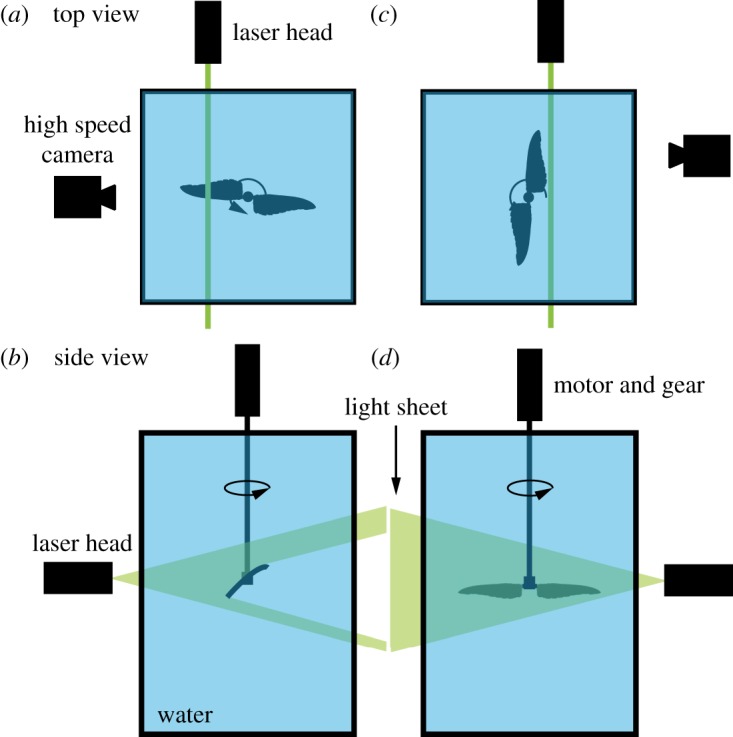
Top and side views of the experimental schemes that recorded the flow over the wing at *α*=30°: (*a*,*b*) stations along the span; (*c*,*d*) in the near-wake area behind the wing.

The entire mechanical assembly of the flapper was mounted on a six-component force and moment transducer (MINI40, calibration SI-20-1 by ATI Industrial Automation, Apex, NC, USA) that recorded the aerodynamic loads acting on the wings (figure 1*c*). The transducer’s dynamic range was 60 N in lift and 1 Nm in torque. A real-time control unit (National Instruments cRIO-9022, Austin, TX, USA) was used to sample the transducer’s analogue signals, which were recorded simultaneously at 10 kHz. The aerodynamic lift, *L*, and torque due to drag, *Q*, of one wing are referenced to $\frac{1}{2}\rho S{\dot{\theta }}_{\infty }^{2}R_{2}^{2}$ and $\frac{1}{2}\rho S{\dot{\theta }}_{\infty }^{2}R_{3}^{3}$, respectively, yielding the dimensionless lift and torque coefficients, *C*_L_ and *C*_Q_, respectively. *S*,*R*_2_ and *R*_3_ are the complete wing area and the radii of second and third moments of area, respectively ([34], table 1). A description of how the aerodynamic loads were extracted from wing kinematic parameters and load measurements is provided in appendix A. The wings were tested at various installation angles (hereafter angle of attack, *α*), defined as the physical angle between the wing’s root chord and the horizontal plane (flapping plane; figure 2*b*), ranging between 5° and 35° at 5° increments. To improve the signal-to-noise ratio, each time-accurate load acquisition was repeated 50 times (*n*=50).

The time-averaged aerodynamic coefficient for each individual load measurement *i* (1≤*i*≤*n*), is defined as

2.2 $${\bar{C}}_{*,i}=\frac{1}{T}\int_{0}^{T}C_{*,i}(\tau )\;d\tau ,$$

where *={*L*,*Q*}, *T* is the downstroke duration (table 1) and *τ* is the integration variable. Time-averaged aerodynamic coefficients are subsequently defined as

2.3 $${\bar{C}}_{*}=\frac{1}{n}\sum_{i=1}^{n}{\bar{C}}_{*,i}.$$

A 30 s pause was included before each measurement to create an essentially quiescent flow at the beginning of the following wing downstroke motion. Throughout our time-accurate aerodynamic load measurements, a low-pass Butterworth filter was applied offline with a cut-off frequency of 40 Hz.

### 2.4. Flow field measurements

Two schemes of flow field measurements were applied in order to analyse the flow field over the printed model wing at constant span stations (figure 2*a*,*b*) and behind the wing in the near-wake section (figure 2*c*,*d*). Following the first scheme, to investigate the flow over the wing root, middle and tip, the in-plane velocity field was recorded at *z*=*r*/*R*=0.25, 0.5 and 0.75 (figure 1*d*). For better image quality, the flapping frequency was reduced relative to the aerodynamic loads measurements, and therefore, due to similarity constraints, the wing pair was tested in pure water (table 1). In order to analyse the high lift flow mechanisms developing along the downstroke of a hummingbird wing, we focused our efforts to acquire high-fidelity flow field measurements at a single, relatively high, biologically relevant angle of attack, *α*=30° [17,21,22,41,42] (for further explanation as to why this angle of attack was chosen see Results, Flow field section below). The flow fields were acquired using a PIV system (Dantec Dynamics A/S, Skovlunde, Denmark). It consisted of a dual-cavity 30 mJ Nd:YLF laser with a repetition rate of up to 10 Hz, a 4M pixel resolution 12-bit CCD camera (FlowSense EO) and a programmable timing unit. The laser-head was mounted on a platform at the front of the water tank (figure 2*b*,*d*). A cylindrical lens was used to create a thin light sheet of approximately 2 mm in thickness, and the double exposure camera equipped with a 50 mm lens was mounted on a tripod perpendicular to the laser sheet. This set-up yielded a maximal field of view of approximately 210×156 mm, capturing the flow behind the wing along the entire wing span, as shown in figure 2*c*,*d*. Silver-coated, hollowed, glass spheres with a mean diameter of 10 μm (Dantec Dynamics A/S, Skovlunde, Denmark) were used as seeding particles.

During each wing motion, high-speed image sequences were recorded at several angular wing positions throughout the motion. Using an optical switch (YH03NCT8, Wenglor, Tettnang, Germany), a synchronization pulse was fired to the camera and laser-head at these specific angular positions. In order to keep the same camera calibration and illumination settings, the initial angular position of the wing was modified to allow for image capturing at a fixed position with respect to the camera. To improve the signal-to-noise ratio, each test condition was repeated 50–80 times. Similar to the acquisition of the aerodynamic loads, the wing was paused at the end of each wing downstroke in order to establish an essentially quiescent flow at the beginning of each successive measurement. The PIV processing was conducted using DynamicStudio v. 3.4.1 (Dantec Dynamics A/S, Skovlunde, Denmark). The two-dimensional PIV procedure yielded two in-plane velocity components, which were computed from the cross-correlation of pairs of successive images with 50% overlap between the interrogation domains. The seeding density allowed for high-quality vector fields with interrogation windows of 32×32 pixels (spatial resolution of approx. 1.6 mm).

The dimensional unsteady velocity field is denoted as $\text{U}=\{U,V,W\}=(1/n)\Sigma_{i=1}^{n}{\text{U}}_{i}$, where **U**_*i*_ is the flow field that was recorded at the *i*th measurement (1≤*i*≤*n*). *U*,*V* and *W* are the velocity components in the *X*,*Y* and *Z* directions, respectively (figure 1*d*). The tangential velocity at each span station is $U_{∥}(z)=\dot{\theta }R(z+\eta )$ (figure 1*d*). The dimensionless velocity field in the wing’s frame of reference is defined as **u**={*U*−*U*_∥_,*V*,*W*}/*U*_∥_={*u*,*v*,*w*}. The near-wake velocity fields refer to the wing tip velocity, u*=**u**/*U*_*tip*_, where $U_{\mathrm{tip}}=\dot{\theta }R(1+\eta )$. For statistical analysis, the velocity fluctuations are defined using the standard definition referring to the wing tip velocity at mid-downstroke, $\bar{\zeta^{\prime 2}}=(1/n)\Sigma_{i=1}^{n}{\left[(\zeta_{i}-\zeta )U_{∥}/U_{\mathrm{tip}}^{90^{\circ }}\right]}^{2}$, where *ζ*=*u*,*v* or *w*; it is understood that *ζ*_*i*_ denotes *ζ*’s *i*th measurement. We define a standard deviation operator for two components of the vector field as $\sigma_{u_{1}u_{2}}={\left[\frac{1}{2}\left(\bar{u_{1}^{\prime 2}}+\bar{u_{2}^{\prime 2}}\right)\right]}^{1/2}$.

## 3. Results

### 3.1. Aerodynamic loads

**Figure 3.**
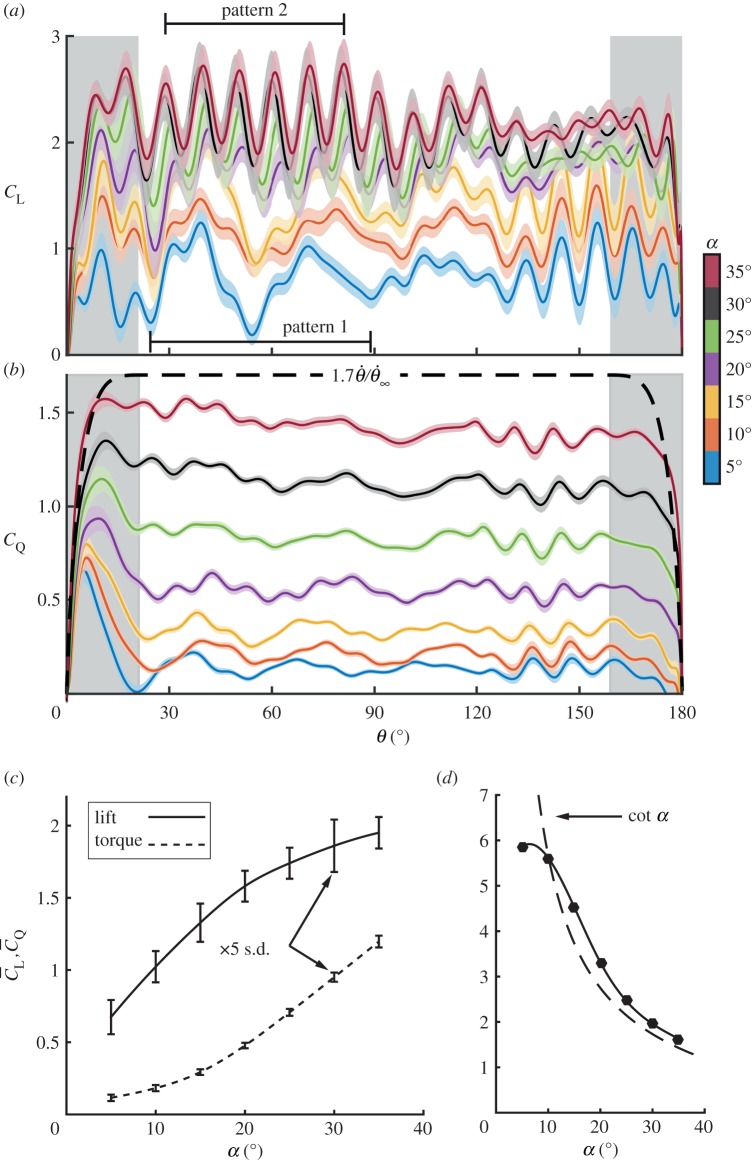
Time-accurate aerodynamic coefficients along the downstroke at all tested angles of attack: (*a*) lift (*b*) torque due to drag. Solid lines denote ensemble-averaged values; shaded regions represent the standard deviation (s.d.) value at each angular position. The dashed line in (*b*) represents the wing angular velocity profile along the downstroke. (*c*) Time-averaged aerodynamic coefficients at different angles of attack. Error bars represent the time-averaged standard deviation of measurements, magnified five times, for clarity. (*d*) Lift-to-torque ratio, compared to Polhamus leading-edge vortex geometric model of cot⁡α [43], denoted by a dashed line.

The time-dependent development of the aerodynamic coefficients, *C*_L_ and *C*_Q_, is presented in figure 3*a*,*b*, respectively, for various angles of attack. The standard deviation (s.d.) of the aerodynamic load measurements is represented by the shaded region on top of the mean value for each one of the cases. At *α*≤20°, lift levels increased monotonically along with the angle of attack, while, at *α*≥25°, lift production reached saturation. Interestingly, unlike the lift production over rotating wings at low Reynolds numbers [26], the lift coefficient did not reach a steady state during the downstroke (figure 3*a*). Two oscillatory patterns were measured; pattern 1 corresponding to 5°≤*α*≤15°, where damped oscillations appear, and pattern 2 at 20°≤*α*≤35°, where oscillations of higher frequency persist throughout most of the downstroke. This phenomenon will be further discussed below. Unlike *C*_L_’s development along the downstroke, the results show a distinct correlation between the torque coefficient, *C*_Q_, and the angular velocity profile (figure 3*b*).

The time-accurate development of the aerodynamic loads was time-averaged in order to efficiently quantify all of the aerodynamic characteristics of the downstroke (as presented in equations (2.2) and (2.3)). The time-averaged aerodynamic coefficients, ${\bar{C}}_{L}$ and ${\bar{C}}_{Q}$, are presented in figure 3*c*, showing similar trends as were measured in previous studies of dried hummingbird wing models [16,22] and of insect wings with a similar aspect ratio (table 1, [44,45]). For clarity, error bars represent the standard deviation magnified five times, illustrating the high repeatability of the results. While lift increased monotonically at 5°≤*α*≤20°, as suggested from the time-accurate lift coefficients (figure 3*a*), the time-averaged torque coefficient, ${\bar{C}}_{Q}$, grew parabolically with the angle of attack, yielding a $\max\{{\bar{C}}_{L}/{\bar{C}}_{Q}\}$ of about 6 at *α*=5° (figure 3*d*).

### 3.2. Flow field

The flow fields over the *C. anna*’s wing model were acquired at *α*=30°. Such angle is at the higher end of hummingbirds’ operational angles of attack [17,21,22,41,42]. This allowed us to analyse coherent flow features over the wing, and qualitatively relate the flow fields to lower angles according to pattern 2 (as low as *α*=20°, figure 3*a*).

**Figure 4.**
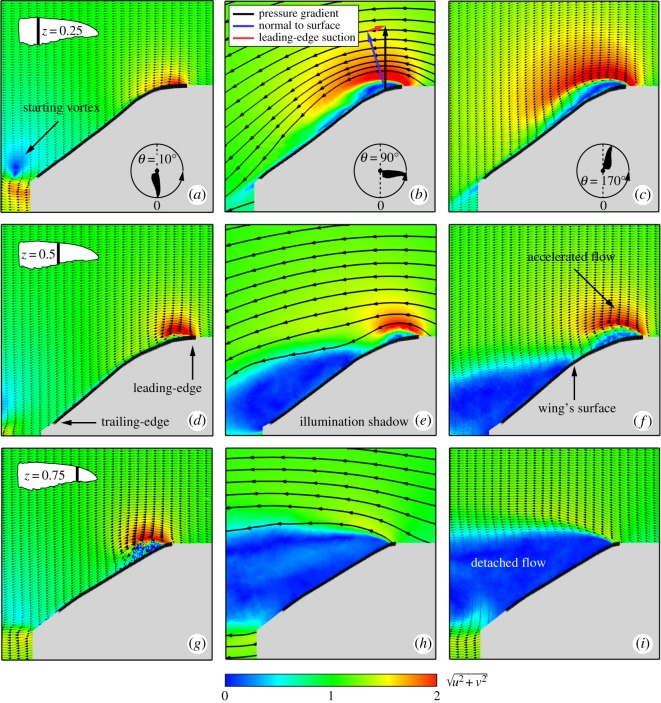
Flow field development at stages throughout the downstroke at *α*=30°. Rows are arranged by ascending span location: (*a*–*c*) *z*=0.25, (*d*–*f*) *z*=0.5 and (*g*–*i*) *z*=0.75. Columns are arranged by ascending angular position: *θ*=10° (*a*,*d*,*g*), *θ*=90° (*b*,*e*,*h*) and *θ*=170° (*c*,*f*,*i*). The colour scheme describes the in-plane velocity magnitude. Column (*b*,*e*,*h*) depicts the streamlines over the wing. The solid black line depicts the wing’s position, and the shaded grey area is the illumination shadow region behind it.

Representative flow fields for several angular wing positions along the downstroke are presented in figure 4. The dimensionless magnitude of the in-plane velocity, $\sqrt{u^{2}+v^{2}}$, is described by the background colour. At the early stages of wing acceleration, (e.g. *θ*=10°; figure 4*a*,*d*,*g*), traces of a starting vortex were found throughout the span behind the trailing-edge as it was shed into the wake. In the vicinity of the leading-edge, an accelerated flow region was apparent, covering about 0.2*c* at *z*=0.25, where *c* is the local chord length. The chordwise extent of this region monotonically grew throughout the wing’s motion (figure 4*a*) along the wing span (figure 4*g*). From mid-downstroke (*θ*=90°) onwards, the flow fully detached over the wing tip (figure 4*h*,*i*), indicating an unsteady flow in this area. A large portion of the mid-span cross section indicated a massive detached flow region as the wing reached mid-downstroke (figure 4*e*, *θ*=90°). Several factors contribute to this outcome, including the high spanwise velocity component at the medial portion of the wing [24] and the low tangential velocity (which translates to a low local chord-based Reynolds number). In addition, the leading-edge radius, which is as large as $R_{l.e}/\bar{c}=0.038$, contributes to the fact that in *z*=0.25, the flow remains attached throughout the chord during the entire downstroke. It is, therefore, not surprising that the flow field throughout the remainder of the downstroke remains similar in nature to that which was recorded at mid-downstroke (figure 4*c*,*f*,*i*).

The lift-to-drag ratio was previously used to assess whether a leading-edge vortex mechanism is present [43]. In such scenarios, the flow separates from a sharp leading-edge, causing elimination of or a severe drop in leading-edge suction. This yields a net force which is normal to the wing surface, and therefore, a geometric relation between lift and drag forces, where L/D=cot⁡α [43]. This relationship was originally suggested for delta wings by Polhamus and was shown to be applicable in describing the lift-to-torque ratio of revolving insect wings [45]. A comparison between the geometric model and the results obtained for the *C. anna*’s wing model are presented in figure 3*d*. The results indicate that Anna’s hummingbird’s lift-to-torque ratio followed cot⁡α for *α*>10°, which suggests the presence of similar flow mechanisms as were found for insects. However, the lift-to-torque ratio measured for Anna’s wing outperformed that of insects for various angles of attack, i.e ${\bar{C}}_{L}/{\bar{C}}_{Q}>\cot\alpha$. Streamlines extracted from the flow field measurements are used to qualitatively explain the improved performance of Anna’s wings (figure 4*b*,*e*,*h*). At the proximal span station, the local pressure gradient did not act normal in the wing surface (black arrow, figure 4*b*). This implies the development of a leading-edge suction force (red arrow), resulting in a lower ${\bar{C}}_{Q}$ (and therefore ${\bar{C}}_{L}/{\bar{C}}_{Q}>\cot\alpha$). Further along the span, the leading-edge suction force is diminished as the leading-edge radius is much smaller (figure 4*e*), and at *z*=0.75 the flow is fully detached (figure 4*h*). The leading-edge radius is not the only factor that influences leading-edge suction and further studies are planned to determine the role of wing camber, twist and wing surface texture on this aspect.

**Figure 5.**
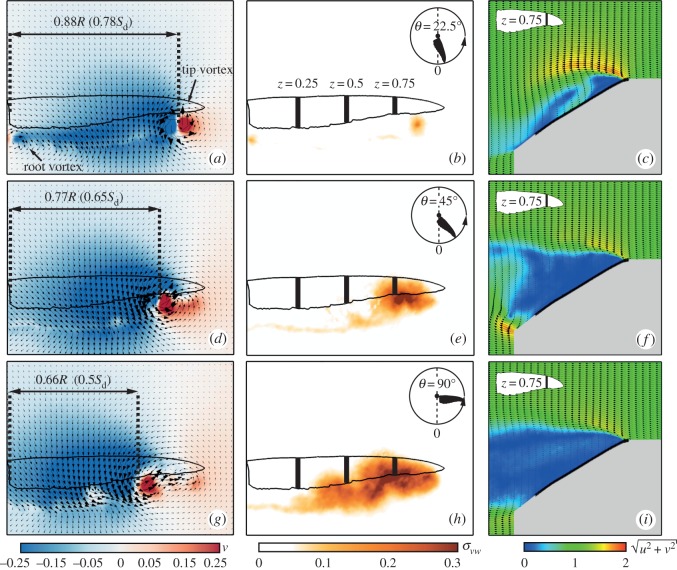
Near-wake flow fields measured at *α*=30°. Rows are arranged by ascending angular position: (*a*–*c*) *θ*=22.5°; (*d*–*f*) *θ*=45°; (*g*–*i*) *θ*=90°. Column (*a*,*d*,*¡textit¿g*¡/textit¿) depicts the near-wake flow field. The colour scheme denotes the ensemble averaged vertical velocity component, *v*; the flow fields standard deviation, *σ*_*vw*_, is presented in column (*b*,*e*,*h*). Wing contour and the measured span stations are shown for reference; the flow fields at *z*=0.75 are presented in column (*c*,*f*,*i*); see figure 4 for panel descriptions.

One of the flow quantities associated with upward (or, positive) lift is the effective disc area. This area is denoted by a region of downward (or negative) vertical velocity, *v* (background colour in figure 5*a*,*d*,*g*). In these figures, the effective discs of representative stages along the downstroke are referenced to the wing disc area (*S*_d_=*π*[(*R*+*Rη*)^2^−(*Rη*)^2^]). At *θ*=22.5°, the effective disc area is restricted by two coherent vortices, one at the wing root and one at its tip (figure 5*a*,*b*), similar to vortices observed in *in vivo* flow field measurements [20]. Seventy-eight per cent of the wing disc at this stage contributed to the effective disc area (figure 5*a*), while at later stages this area shrank to 65% at *θ*=45° (figure 5*d*) and 50% at *θ*=90° (figure 5*g*). At mid-downstroke, the wing tip vortex broke down. This phenomenon is described by the growing flow fluctuations within that flow region (figure 5*h*). This vortex breakdown started in the vicinity of the wing tip and progressed towards the medial portion of the wing, as it reached *z*=0.5 at *θ*=45° (figure 5*e*) and *z*=0.25 at *θ*=90° (figure 5*h*).

## 4. Discussion

### 4.1. Survival of the leading-edge vortex

**Figure 6.**
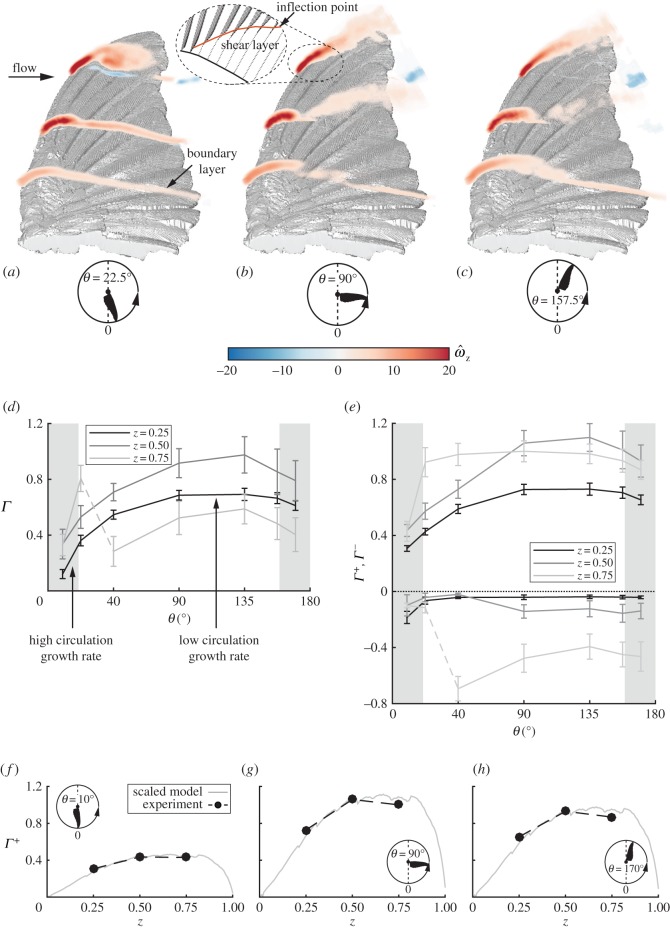
Vorticity contours measured at *α*=30° over the wing depicting the spanwise vorticity concentration development at (*a*) *θ*=22.5°, (*b*) *θ*=90° and (*c*) *θ*=157.5°. A magnification of the tangential flow field in the vicinity of the leading-edge is presented for the distal span station (*z*=0.75) in (*b*), where the red line denotes the inflection points. (*d*) Net circulation; (*e*) positive and negative circulation. Circulation distribution along the span (scattered dots), corresponding to Ellington’s dynamic stall model [35] at (*f*) *θ*=10°, (*g*) *θ*=90° and (*h*) *θ*=170°. The model was scaled to produce the same integrated circulation along the span as the measured circulation.

The lift-to-torque ratio, which is proportional to cot⁡α (figure 3*d*), indirectly suggests that the main lift mechanism over the *C. anna* wings is the leading-edge vortex [45] or, otherwise, a concentration of spanwise vorticity in the vicinity of the wing’s leading-edge. The development of the dimensionless spanwise vorticity along the downstroke, ${\hat{\omega }}_{z}=\omega_{z}\bar{c}/U_{\mathrm{tip}}^{90^{\circ }}$, where *ω*_*z*_ is the dimensional vorticity, is presented in figure 6*a*–*c*. As the wing reached its constant angular velocity stage, at *θ*=22.5° (figure 6*a*), a large concentration of clockwise vorticity formed over the leading-edge, growing throughout the span from the root towards the wing tip. An attached boundary layer formed behind the vorticity concentration at both *z*=0.25 and *z*=0.5. At *z*=0.75, flow detachment was apparent, where the spanwise vorticity structure and the boundary layer itself rose above the wing surface. The detached flow region is characterized by growing levels of flow fluctuations which were highest at the distal-most wing stations (figure 5). Along the downstroke, the region of high-flow fluctuation levels expanded medially (figure 5*h*). Further downstream to the flow detachment area, a shear layer formed which is characterized by an inflection point in the tangential velocity profile (figure 6*b*; the red line describes the inflection point over the wing).

The spanwise vorticity structure presented above differs from the leading-edge vortex associated with flow over wings at considerably lower Reynolds numbers [24,25,28,46]. At characteristic Reynolds numbers of several hundred, the leading-edge vortex is almost circular (aspect ratio of about 1) and extends over about half of the local wing chord [24,25,28]. The present results from *C. anna* wings show that the spanwise vorticity structure covers a maximum of about one-third of the local chord length at mid-span and *θ*=157.5° with an aspect ratio ranging between 8 and 3.3 at the root and medial span stations, respectively (figure 6*c*). The structure of the spanwise vorticity concentration, found over the leading-edge, is similar to that previously found over rufous hummingbirds [19]. Moreover, the flow fluctuation levels within the spanwise vorticity structure over the wings of Anna’s hummingbird are considerably higher than those over insect wings, as in the latter, the leading-edge vortex was found to remain steady (essentially zero flow fluctuations).

In order to quantify the contribution of the leading-edge spanwise vorticity to the wing lift, the dimensionless circulation was calculated by integrating the dimensionless vorticity fields [46]. The development of the net circulation (*Γ*) over the three measured span-stations is presented in figure 6*d*. During wing acceleration (*θ*<22.5°), high circulation growth rates were measured. As reported before [46], this phenomenon can be explained by the low vorticity convection towards the wing tip, because spanwise velocity has not developed considerably at this stage of the downstroke. Later on, spanwise velocity grows, causing the accumulated vorticity to convect towards the tip and slow down the circulation growth rate (figure 6*d*).

At the end of the acceleration stage (22.5°<*θ*<45°), the net circulation over the distal span station (*z*=0.75) abruptly dropped, as marked by the dashed line in figure 6*d*. To better explain this phenomenon, the net circulation was decomposed into its positive (spanwise), *Γ*^+^, and negative, *Γ*^−^ parts (see figure 6*e*). Naturally, *Γ*^+^ is associated with spanwise vorticity production over the leading-edge. *Γ*^−^ and its characteristically high standard deviation are associated with flow fluctuations. It is apparent that the large detached flow region in the vicinity of the wing tip induced high values of negative circulation resulting in the abrupt drop in net circulation (figure 6*d*). At mid-downstroke (*θ*=90°), flow field measurements over the mid-span of the wing (*z*=0.5) have also suggested the development of a large detached flow area forming behind the leading-edge vorticity structure (figure 4*e*). It is noteworthy that despite the abrupt growth of the negative circulation (in magnitude), as presented for *θ*=45°, the net circulation kept its monotonic growth until the wing reached *θ*=135° (figure 6*d*). This outcome emphasizes the significance of the vorticity structure forming over the leading-edge and the strength of the positive circulation (*Γ*^+^) in that area.

By referring to previous studies’ circulation measurements and specifically to their corresponding wing tip velocities and chord lengths, comparisons can be made to the dimensionless circulation presented in figure 6*d*,*e*. Good agreement to similar *in vivo* dimensionless flow field measurements of a rufous hummingbird is evident, which may suggest a similar vorticity mechanism under natural free-flight settings [19]. Interestingly, dimensionless circulation values are also similar to those measured over insect wing models at different Reynolds regimes, suggesting that the vorticity concentration found over the wing model of *C. anna* performs similarly to an attached leading-edge vortex [47]. This similarity encouraged us to compare the presented results to Ellington’s dynamic stall model (figure 5*f*–*h*), which was originally formulated in order to quantify the leading-edge vortex that formed over insect wings at Reynolds numbers between a hundred and a few thousand [35]. It suggests that circulation associated with upwards lift, *Γ*^+^, is proportional to *zc*, where *z* is the spanwise location and *c* is the corresponding wing chord length. For each stage along the downstroke, a proportional scale factor was set such that the measured circulation matched that of the model in order to analyse its distribution along the span. Applying this model to *Γ*^+^ in the presented analysis showed a remarkable match, suggesting that the spanwise vorticity concentration over *C. anna*’s wings yields positive circulation, similar to the one that developed over wings of much lower Reynolds numbers. However, the net circulation was not comparable to Ellington’s dynamic stall model. Therefore, the presented spanwise vorticity structure that was measured over the wings of *C. anna* will be hereafter dubbed a *leading-edge bubble*.

### 4.2. Flow structures

**Figure 7.**
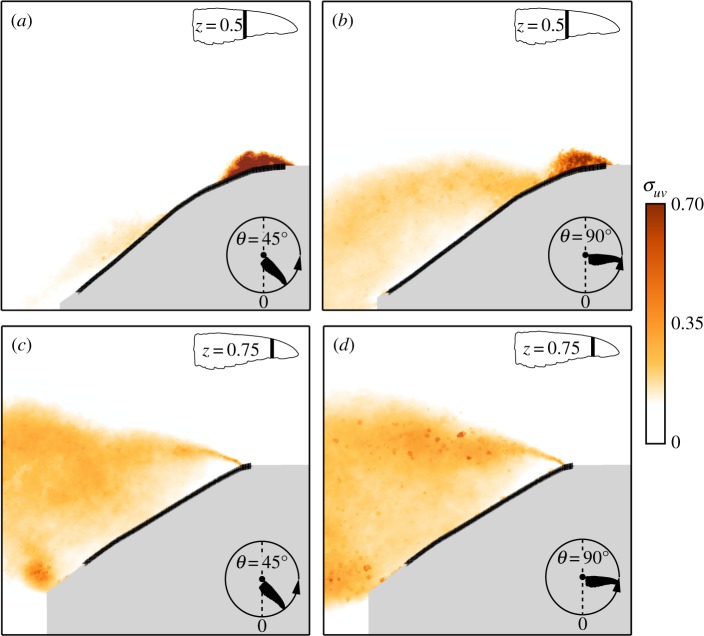
Flow field standard deviation, *σ*_*uv*_, at representative span stations and angular positions along the downstroke, measured at *α*=30°: (*a*) *z*=0.5, *θ*=45°; (*b*) *z*=0.5, *θ*=90°; (*c*) *z*=0.75, *θ*=45°; (*d*) *z*=0.75, *θ*=90°.

During the downstroke, the flow field over *C. anna*’s wings, as was already shown above, is highly unsteady (figure 5). Flow fluctuation levels over the wing at different span stations reached values as high as *σ*_*uv*_=0.7 (figure 7). At wing stations where the flow is mostly attached to the wing (i.e, *z*=0.5, and *θ*=45°, figure 7*a*), high standard deviation values are mostly constrained within the leading-edge bubble. Detached flow regions are characterized by high values of flow fluctuations (figure 7*b*–*d*), but these are not restricted only to the vicinity of the leading-edge. Interestingly, the lift standard deviation at *α*=30°, matched the standard deviation of the integral of net circulation (*Γ*) along the span, stressing the relation between lift and net circulation and not only to that which originated from *Γ*^+^.

**Figure 8.**
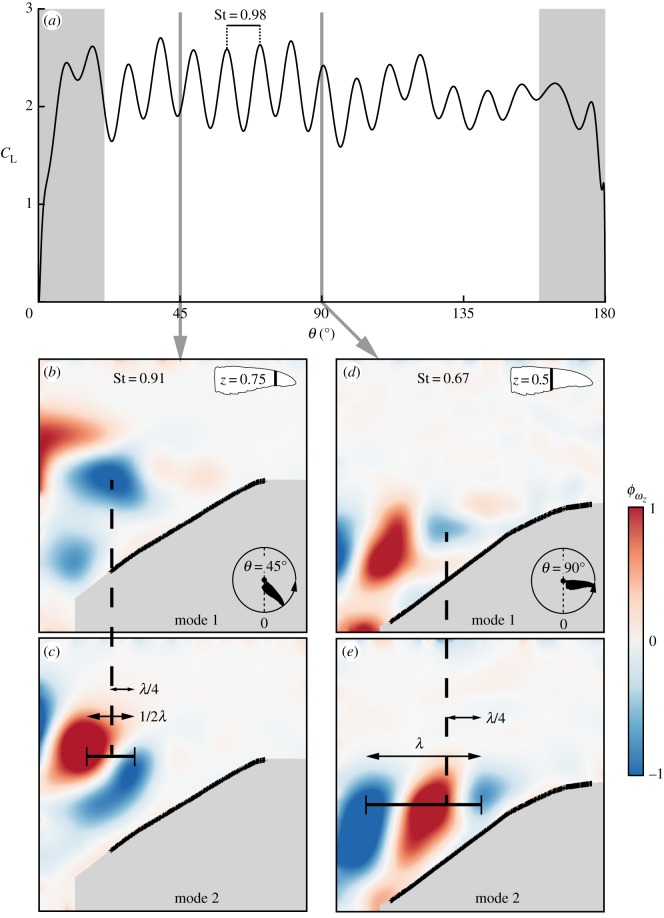
Modal inspection of the flow fields, measured at *α*=30°, and links to aerodynamic forces. (*a*) *C*_L_ at *α*=30°. The spanwise vorticity fields of the two strongest modes (referenced to the highest vorticity value) at representative span stations and angular position along the downstroke: (*b*,*c*) *z*=0.75 and *θ*=45°; (*d*,*e*) *z*=0.5 and *θ*=90°. *λ* is the structure shedding wavelength.

The time-accurate load measurements indicate a certain frequency which is dominant during the downstroke (figure 8*a*). The typical dimensionless frequency of these undulations (Strouhal number) is St=0.98, where

4.1 $$\text{St}=\frac{f_{u}\bar{c}}{U_{\mathrm{tip}}^{90^{\circ }}}$$

and *f*_*u*_ is the dimensional frequency of these undulations. In order to investigate this aspect, proper orthogonal decomposition (POD–snapshot method, [48]) was applied to the spanwise vorticity fields. The outcome of this analysis is the spatial modes, *ϕ*_*ω*_*z*_*i*_, and their respective energy levels. The first two spatial modes of the dimensionless vorticity field, sorted by their energy levels, are presented in figure 8. The two vorticity modes are phase-shifted by a quarter of a wavelength between each mode pair (figure 8*b*–*e*). Previous studies have associated such a phase shift with two-dimensional vortex shedding [49,50]. Naturally, the flow field over the *C. anna* rotating wings is three-dimensional, due to the spanwise pressure gradient along the span. However, it can be inferred from the phase shift between the two dominant spatial modes that the vorticity shedding is probably governed by two-dimensional flow mechanisms rather than three-dimensional ones. If this description is accurate, the high-level flow fluctuations should be associated with the shear layer in each span station as the origin of flow instability [51]. Future research is required to further explore this aspect.

As POD does not yield the respective frequency of the dominant spatial modes, these were estimated using the wavelength as the appropriate length scale and the convective velocity between two adjacent vorticity concentrations. At *θ*=45° and *z*=0.75 (figure 8*b*,*c*), this analysis yields a dimensionless frequency of St=0.91, in good agreement with the dimensionless frequency that characterized the direct lift measurement (St=0.98, figure 8*a*). This suggests that these flow fluctuations originated from the vorticity concentrations which were shed from the leading-edge bubble further downstream. At *θ*=90° and onwards, more frequencies are evident (figure 8*a*); indeed, at *z*=0.5, the dominant Strouhal number in the flow field is St=0.67 (figure 8*d*,*e*). It is likely that this diversity of frequencies is due to different sheer layer thicknesses and convective velocities at each spanwise station; however, this should be confirmed in a dedicated study.

### 4.3. Hovering performance

Hovering is a demanding type of flight as weight support is solely a function of the flight muscles’ capabilities. To assess the effects of the oscillating aerodynamic loads and flow fluctuations on wing performance, the downstroke time-averaged aerodynamic coefficients were used to estimate the entire wingbeat (downstroke and upstroke) aerodynamic characteristics during hovering. The required time-averaged lift coefficient (given per wing) to support the bird’s body weight is

4.2 $${\bar{C}}_{L_{b}}=\frac{m_{b}g}{\rho_{a}S_{b}{\left(2\pi f_{b}R_{2_{b}}\right)}^{2}},$$

where m_*b*_=4.6±0.22 g (mean ± s.d.) is the *C. anna*’s body mass [37], *g* is the gravitational acceleration, *ρ*_a_ is the air density at sea level and *f*_b_ is the wingbeat frequency (*f*_b_=40 *Hz*, [39]). *S*_b_ and *R*_2_b__ are the bird’s wing area and radius of the second moment of area, respectively.

Flow field measurements indicate that 66% [19,20,52] to 75% [21] of weight support is provided by the downstroke motion of the *C. anna* wing. Therefore, the entire natural wingbeat time-averaged lift (${\bar{C}}_{L_{\mathrm{wb}}}$) can be indirectly estimated from the downstroke lift production, which was measured in the current set-up. For equal downstroke and upstroke durations [22,41],

4.3 $${\bar{C}}_{L_{\mathrm{wb}}}=\frac{{\bar{C}}_{L_{\mathrm{downstroke}}}+{\bar{C}}_{L_{\mathrm{upstroke}}}}{2}=\frac{h^{-1}{\bar{C}}_{L}}{2},$$

where *h* is a proportional factor accounting for the downstroke contribution to the overall wingbeat lift production (0.66≤*h*≤0.75). A sensitivity analysis showed that the presented ${\bar{C}}_{L_{\mathrm{wb}}}$ is practically insensitive to uncertainties between the upstroke and downstroke durations [17].

In order to assess the biological conditions in which Anna’s hummingbirds hover, aerodynamic lift is required to support the bird’s weight; therefore, ${\bar{C}}_{L_{b}}={\bar{C}}_{L_{\mathrm{wb}}}$. According to equation (4.3), the downstroke wing motion is required to yield a lift coefficient of ${\bar{C}}_{L}=2h{\bar{C}}_{L_{b}}$ in order to provide sufficient lift during the entire wingbeat. Under the conditions described above, the downstroke time-averaged lift coefficient has to reach a value which is as high as ${\bar{C}}_{L}=1.44\pm 0.23$ due to the uncertainties associated with *h* and m_b_; Other uncertainties were found to be negligible [7]. This lift coefficient spectrum corresponds to an average downstroke angle of attack, $\bar{\alpha }=17.4\pm 4.7^{\circ }$ (figure 3*c*). These results nicely match previous studies performed on *C. anna*’s wings [22] and on rufous hummingbirds [17] and confirm our approach and methodology.

**Figure 9.**
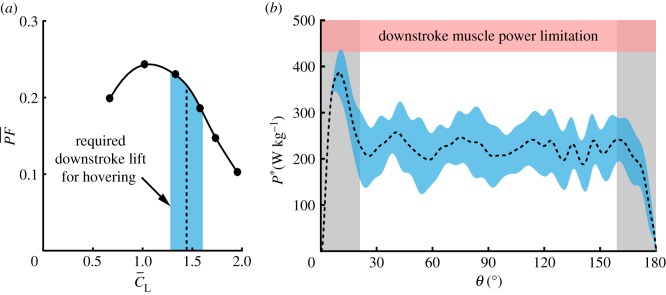
Performance characteristics of *C. anna*’s wing. (*a*) $\bar{\text{PF}}$ versus ${\bar{C}}_{L}$. The dashed line marks the time-averaged downstroke lift coefficient required to support the weight of a hovering hummingbird. (*b*) Time-accurate specific power throughout the downstroke. The specific power is interpolated to describe hovering conditions (dashed line). The shaded blue regions mark the uncertainties according to the variation of m_b_ and *h*. The shaded red region represents the lower downstroke specific power muscle limitations [4].

A key factor enabling hummingbirds to sustain hovering flight is their flight muscle power output. The power factor ($\bar{\text{PF}}$), which represents Anna’s hummingbird flight efficiency, is defined as the inverse of the aerodynamic power required to support a unit of weight (by a wing pair),

4.4 $$\bar{\text{PF}}=\frac{{\text{MTU}}_{\mathrm{ref}}}{h^{-1}\int_{0}^{T}Q(\tau )\dot{\theta }(\tau )\;d\tau },$$

where *M* is the weight to be lifted and $U_{\mathrm{ref}}=\sqrt{2M/\rho S_{d}}$ [53]. The linkage between aerodynamic lift and drag (or, torque; figure 3*d*) allowed us to assume the same *h* to estimate the downstroke contribution to the overall aerodynamic torque and power. It is understood from equation (4.3) that for hovering flight, the dimensional downstroke time-averaged lift is $\bar{L}=2hM$. Under these conditions (see dashed line in figure 9*a*), Anna’s hummingbird hovers at lower $\bar{\text{PF}}$ then the wing’s maximal capability during the downstroke. Such hovering operational condition can be explained by Anna’s hummingbird’s behavioural repertoire as described by Williamson [6]. Throughout the breeding season, along with the necessity to fly between flowers during foraging, males are highly aggressive towards other hummingbirds and are involved in prolonged chases and frequent patrols at different parts of their territories. These territories can stretch over an area of 0.02–0.04 *km*^2^ [54]. Under these ecological settings, natural selection may operate on the bird’s behaviour and energy and time budgets by optimizing flight performance, taking into account both forward and hovering flights [17,22], which may lead to reduced hovering efficiency, but improve overall performance and fitness.

We use our dimensionless results in order to estimate *C. anna*’s dimensional energetics. The hovering muscles’ aerodynamic power output during the downstroke, *P*, was calculated (multiplied by 2 to refer to a wing pair) using the time-accurate aerodynamic torque due to drag (*C*_Q_):

4.5 $$P=\rho_{a}S_{b}{\left(2\pi f_{b}\right)}^{3}R_{3b}{\left(2\pi f\right)}^{-1}C_{Q}\dot{\theta }.$$

*R*_3b_ is Anna’s hummingbird wing radius of the third moment of area. Assuming that flight muscle mass (m_m_) is 25% of the body mass [31,32], the total wingbeat time-averaged muscle specific power is given by

4.6 $${\bar{P}}_{\mathrm{wb}}^{*}=\frac{h^{-1}\int_{0}^{T}P(\tau )\;d\tau }{2Tm_{m}}.$$

Our experimental calculation suggests that ${\bar{P}}_{\mathrm{wb}}^{*}=86\pm 23\;{W\;\mathrm{kg}}^{-1}$ while hovering at the appropriate angle of attack to sustain hover $\bar{\alpha }$. Our result corresponds well with the specific muscle power found for small- to medium-sized, free-flying hummingbirds, ranging between 76 W kg^−1^ for *S. rufus* and 93 W kg^−1^ for *Eugenes fulgens* [4], results which were associated with perfect elastic storage [55,56]. This outcome is another confirmation of our experimental approach and provides a reliable estimation of the entire wingbeat performance.

Since each wing is accelerated and decelerated along the downstroke, flight muscles must overcome varying power requirements which may be higher than the time-averaged one. Using the time-accurate aerodynamic load measurements, the varying specific power (P*) along the downstroke is presented in figure 9*b* for $\bar{\alpha }$ (*C. anna*’s hovering condition). P* was estimated assuming that the pectoralis consist of two-thirds of Anna’s hummingbird flight muscle mass [33], therefore

4.7 $$P^{*}=\frac{P}{(2/3)m_{m}}.$$

Chai & Millard [4] estimated the maximal wingbeat specific power for different hummingbird species by testing their maximal loading capabilities. Hummingbirds were able to hover under these extreme conditions, lifting almost twice their body mass for a duration of about 0.5 s. For *C. anna*, this duration is equivalent to about 20 wingbeat cycles. To offer a valid comparison to our results, Chai & Millard’s maximal power values were translated to reflect the averaged downstroke specific power using equation (4.6). The shaded red area in figure 9*b* corresponds to the time-averaged downstroke maximal loading capabilities, where the lower limit corresponds to *S. rufus* [4]. As expected, the highest power requirement is during the acceleration stage, when muscles need to overcome the wings inertia. A peak value of 379 W kg^−1^ (mean value) was measured, 70% more than the power requirement during the constant velocity phase. Despite the initial high power requirement, Anna’s hummingbird is believed to operate well within a safety margin throughout most of the downstroke, as was suggested by Chai & Millard [4], allowing plenty of power reserves for aerial manoeuvres (e.g. high-speed courtship flight).

## 5. Conclusion

This study is part of our effort to analyse the aerodynamic characteristics of the flapping wing of *C. anna* throughout its annual cycle. In the present contribution, we concentrated on the baseline wing configuration while laying the foundations of our analysis techniques that will be used to address the flow mechanisms and aerodynamic characteristics of several wing geometries that characterize several stages of the wing moult cycle [14]. The flow field over the wing revealed unsteady spanwise vorticity concentrations that are formed and shed in the vicinity of the leading-edge. Because this spanwise vorticity formation has some similarities to the leading-edge vortex that was found over flapping wings at much lower Reynolds numbers, this phenomenon is dubbed the leading-edge bubble. In this bubble, the flow separates and creates a low pressure region, similar to the one that evolved within the leading-edge vortex. The difference between the two is that in the former, vortex shedding evolves too, introducing more time scales to this flow field (or more frequencies). As this evolution does not reach a steady state (like the one over a steady translating wing), the aerodynamic characteristics remain fruitful (i.e. high enough lift-to-torque ratio and notable lift). At the end of stroke, these vorticity concentrations are being convected thanks to the down-wash and a new leading-edge bubble is being created. The unsteady nature of the leading-edge bubble was revealed by the time-accurate load measurements and nicely matched the detailed spatial analysis of the flow field.

The high fidelity and time-accurate results are in good agreement with power requirements estimated from free-flying hummingbirds. Furthermore, the presented approach allows a detailed and direct investigation of hummingbird wing performance; this approach can be used to further investigate more aspects of the highly complex aerodynamics of hummingbirds and the associated flow mechanisms such as wing reversal and the upstroke of deformed wings.

## Figures and Tables

**Figure 10.**
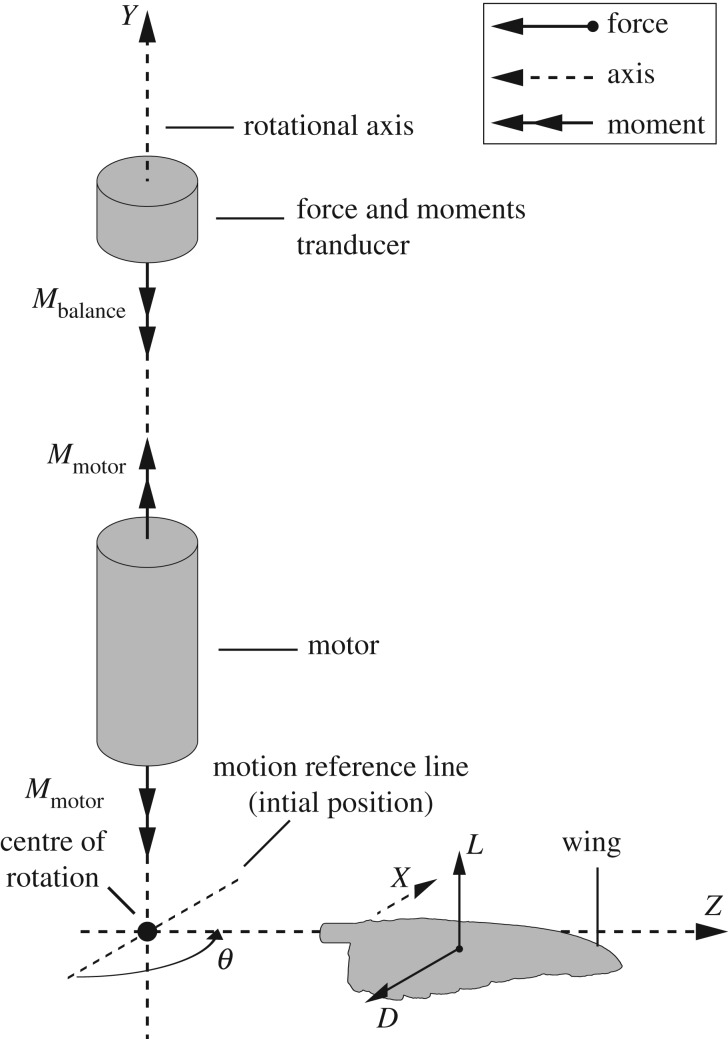
The schematic of the mechanical system. *D* is the drag force acting on the wing.

## Data accessibility

All data underlying this publication are available via Dryad http://dx.doi.org/10.5061/dryad.mr556 [57].

## Appendix A

**A.1. Aerodynamic coefficients calculation**

The measured vertical force (*F*_*y*_, positive upwards) measured by the force and moments transducer is the sum of the following forces acting on one wing:

A 1 $$F_{y}=\underset{\text{Lift}}{\underbrace{L}}+\underset{\text{Wing weight and buoyancy}}{\underbrace{mg+B}}+\underset{\text{Added mass}}{\underbrace{F_{v}}}.$$

To eliminate buoyancy and gravitational acceleration forces, loads measured as the wing was stationary (before each repetition) were subtracted from *F*_*y*_. Added mass effects were estimated using an adaptation of a two-dimensional motion in inviscid fluids to three-dimensions using the blade element theory [27]. Therefore, the added mass in our experimental settings is estimated by

A 2 $$F_{v}=\frac{\rho \pi }{4}\ddot{\theta }\;\sin\alpha \int_{R\eta }^{R(1+\eta )}rc^{2}\;dr,$$

where *r* is the dimensional distance from the wing root and *c* is the dimensional chord length (figure 1*d*). Using this estimation, added mass accounted for less than 1% of the time-accurate aerodynamic loads measured during the acceleration and deceleration stages. Moreover, for the same wing morphology and Reynolds numbers, added mass effects scale proportionally to the aerodynamic loads and therefore would be equivalent to those under biological settings [27]. For these reasons, time-accurate aerodynamic loads are inclusive of the added mass contribution.

The aerodynamic torque, denoted as *Q*, is the torque because drag is acting on the wing. To distinguish the aerodynamic torque, *Q*, from the overall torque measured by the force and moments transducer around the vertical axis (see free-body diagram of the experimental set-up in figure 10), the equation system without external torques is as follows:

A 3 $$\left\{ \begin{array}{l} M_{\mathrm{motor}}=-Q\\ M_{\mathrm{balance}}=M_{\mathrm{motor}} \end{array}\right.\Rightarrow M_{\mathrm{balance}}+Q=0,$$

From equation (.3), we can now deduce the equation with the motor acceleration as

A 4 $$Q=I\ddot{\theta }-M_{\mathrm{balance}},$$

where *I* is the system’s moment of inertia, calculated by the sum value of it is rotating elements; $\ddot{\theta }$ is the angular acceleration.
